# Decoding CD24: Roles of chemoradiotherapy resistance and potential as therapeutic targets

**DOI:** 10.32604/or.2025.059327

**Published:** 2025-05-29

**Authors:** YU HONG, YUNXIANG TANG, WENYAN ZHOU, HANYUE LUO, LINLIN BU, HUI QIU, QIUJI WU

**Affiliations:** 1 School of Medicine, Wuhan University, Wuhan, 430079, China; 2 State Key Laboratory of Oral & Maxillofacial Reconstruction and Regeneration, Key Laboratory of Oral Biomedicine Ministry of Education, Hubei Key Laboratory of Stomatology, Department of Oral & Maxillofacial-Head Neck Oncology, School & Hospital of Stomatology, Wuhan University, Wuhan, 430079, China; 3 Department of Radiation and Medical Oncology, Hubei Key Laboratory of Tumor Biological Behavior, Hubei Provincial Clinical Research Center for Cancer, Zhongnan Hospital of Wuhan University, Wuhan, 430071, China

**Keywords:** Macrophages, CD24, Immune checkpoint, Chemoradiotherapy, Resistance

## Abstract

As a rising immune checkpoint on tumor cells, CD24 is closely related to tumorigenesis and progression. CD24 can directly regulate the malignant behavior of tumor cells and indirectly inhibit the function of immune cells in the meantime, which promotes the immune escape of tumor cells, induces cancer invasion and causes poor prognosis. The basic principle of cancer treatment is to induce cell death and inhibit cell survival. Resistance to chemoradiotherapy is a critical challenge in oncology, which limits the effectiveness of anti-cancer treatments. Many studies have shown a strong association between CD24 and chemoradiotherapy resistance in tumor cells, but the specific mechanism remains unclear. Understanding the mechanisms that CD24 induces chemoradiotherapy resistance may allow us to develop new promising therapeutic strategies to enhance the efficacy of chemoradiotherapy and improve clinical outcomes in the treatment of cancer patients. In this review, we summarized the basic characteristics and functions of CD24, as well as its role in the development of cancer. We focused on the resistance to radiotherapy and chemotherapy mediated by CD24, deciphered fundamental mechanisms and introduced existing clinical studies, with an attempt to propose potential solutions for future explorations.

## Introduction

Radiation therapy and chemotherapy have been well-established as standard treatments for most cancers. However, resistance to these treatments and cancer recurrence remain noticeable challenges in oncotherapy. Understanding the mechanisms behind chemoradiotherapy resistance is a crucial area of cancer research that remains not fully elucidated. During the last decades, it has been recognized that apart from intrinsic characteristics of tumor cells that determine cell demise and clinical outcomes, the complex biological interactions between tumor cells and the matrix which they grow, the so-called tumor microenvironment (TME), also plays significant roles [1]. The TME promotes tumor progression by improving tumor growth, inducing neovascularization, providing hypoxic and acidic conditions, creating immunosuppressive niche and mediating treatment resistance, and therefore becomes a major target for cancer treatments [2,3].

During the process of radiotherapy and chemotherapy, ionizing radiation and cytotoxic agents can affect the interaction between the cellular components and soluble factors of TME, so that the immune progenitor cells in TME fail to differentiate and mature normally or exert their tumor-suppressive effects. On the contrary, the TME is infiltrated by increased regulatory T cells (Tregs), M2 macrophages and myeloid-derived suppressor cells (MDSCs) and other immunosuppressive cells. This leads to immune evasion and acquired drug resistance of tumor cells [4,5].

Tumor-associated macrophages (TAMs) are the most prevalent innate immune cells in the tumor microenvironment [6,7]. Phagocytotic checkpoint expression controls how tumor-associated macrophages behave physiologically. Recent studies have identified several macrophage-associated phagocytic checkpoints, including the CD47/signal regulatory protein α axis (SIRPα), PD-1/PD-L1 axis, CD24/Siglec-10 axis, and major histocompatibility complex class I (MHC-I)/LILRB1 axis, and others [8–10]. Importantly, radiotherapy and chemotherapy induce the expression of some of these checkpoints, thereby inhibiting the phagocytic function, and antigen processing and presentation function of macrophages, compromising the anti-tumor immune response [11]. Through the study of phagocytotic checkpoints, relevant pathways can be targeted to regulate macrophage functions. Following this trail, seeking a delicate balance between immune activation and chemoradiotherapy-induced immunosuppression will be a promising approach to improve cancer treatment.

In recent years, CD24, as a brand-new phagocytic checkpoint, has become a popular potential target for the treatment of cancer and non-neoplastic diseases. CD24 is significantly overexpressed in various types of cancer, including breast cancer, ovarian cancer (OC), and pancreatic cancer. Due to its diverse post-translational modifications, CD24 is associated with tumor development, invasion, and metastasis. The emergence of radiochemotherapy resistance during cancer treatment has also been found to be closely related to CD24. It is considered a potential marker for cancer prognosis and therapy. Furthermore, emerging research has revealed the key role of CD24 in various health conditions, including autoimmune diseases, graft-*vs*.-host disease, and COVID-19.

Here, we introduce the basic characteristics and functions of CD24, as well as its role in the progression of cancer. Subsequently, we discuss the manifestations and mechanisms of radiotherapy and chemotherapy resistance mediated by CD24, summarize the research on cancer immunotherapy targeting CD24, and propose future research directions and solutions for this issue.

## Structure and Expression Regulation of CD24

### Structure of CD24

CD24 was first identified in 1978 and was referred to as a heat-stable antigen due to its heat tolerance [12]. The human *CD24* gene is located on the chromosome 6q21. The *CD24* cDNA has an open reading frame of 0.24 kb and a 3′ UTR of 1.8 kb, and the deletion of the nucleotide in the UTR region affects the stability of *CD24* mRNA [13,14]. CD24 is a glycosyl-phosphatidylinositol (GPI)-anchored glycoprotein [15,16]. It is a membrane bound protein with a small protein core but extensive N-linked and O-linked glycosylation, which are highly variable and histiocytes-specific, resulting in fluctuations in the molecular weight of CD24 protein between 20 and 70 kDa isolated from different tissues or cell types. Glycosylation of CD24 is specific for cell type, which is determined by the cell pool of glycosyltransferases. This is also relevant to cancer, where abnormal upregulation of certain glycosyltransferases will lead to abnormal glycosylation within cancer cells [17]. Such alterations in glycosylation are known to contribute to the malignant phenotype, including potential implications for cell adhesion, immune evasion, and metastasis.

### Regulation of CD24 expression

CD24 is expressed by many cells of the immune system. In general, CD24 is expressed at higher levels in progenitor and metabolically active cells, while is less expressed in terminally differentiated cells. Relevant to this review, CD24 is often overexpressed in human cancers [18,19].

**Figure 1:**
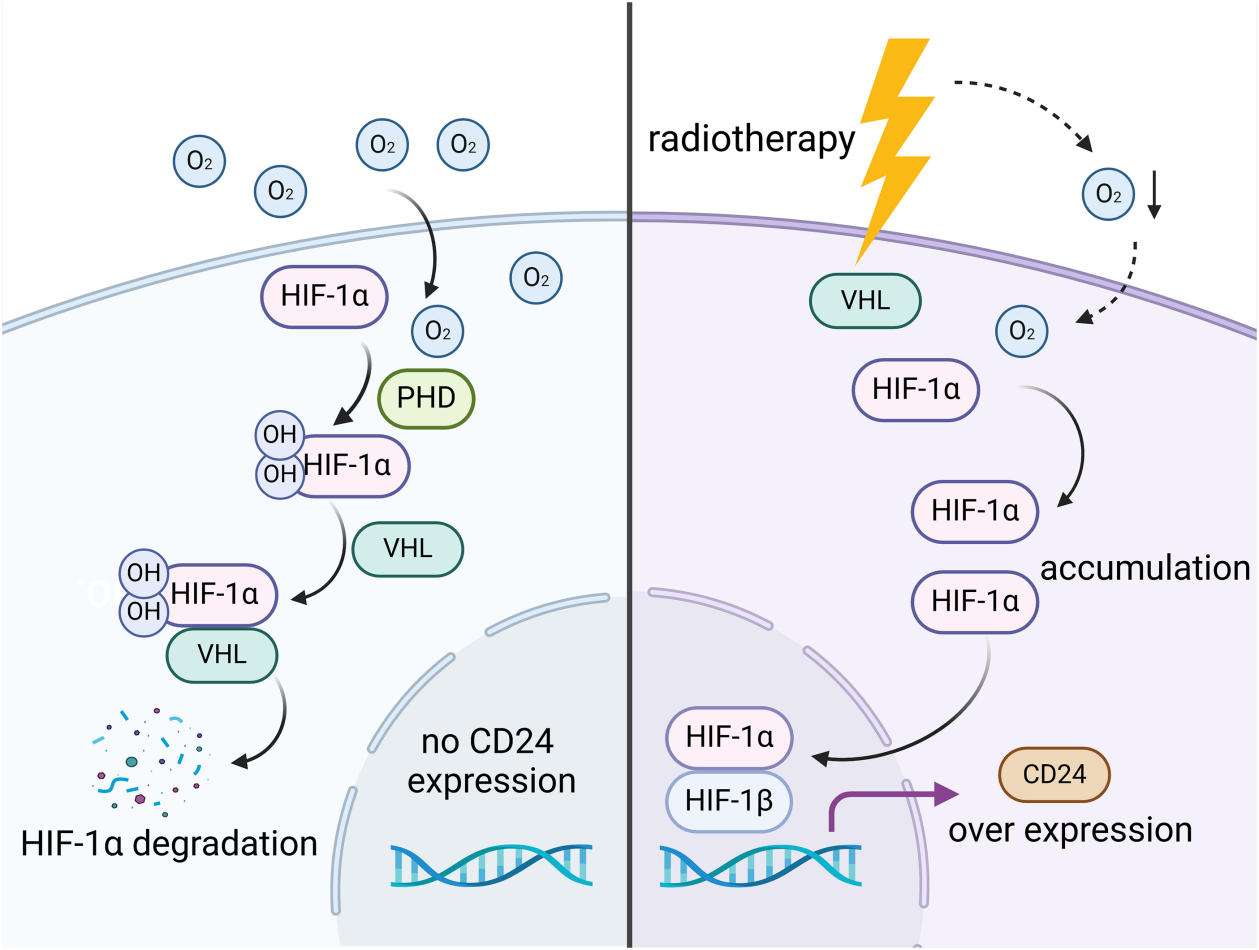
Radiotherapy regulates the expression of CD24. Under normoxic conditions, HIF-1α is hydroxylated in the presence of oxygen, catalyzed by prolyl hydroxylase domain (PHD) enzymes, and then rapidly degraded after binding to von Hippel-Lindau protein (VHL). However, radiotherapy enhances the hypoxic environment in tumor cells, reducing intracellular oxygen levels. This prevents HIF-1α from binding to VHL and undergoing degradation, leading to its accumulation within the cell and subsequent translocation into the nucleus, where it binds to HIF-1β to promote transcription of the CD24 gene, thereby upregulating CD24 expression. Figure created with BioRender.com.

During transcription, hypoxia-inducible factors (HIFs) are induced as transcription factors of CD24 when oxygen supply is limited and oxygen content in the tumor microenvironment is low. Indeed, placing tumor cells in a hypoxic environment promotes CD24 expression through HIFs [20,21]. Regulation of CD24 expression is also associated with tissue-specific response elements in the promoter. In human urothelial carcinoma, CD24 expression is controlled by androgen receptors. Androgen receptors and androgen response elements upstream of the CD24 initiation codon increase CD24 promoter activity when androgen levels rise [22]. In breast cancer, estrogen-reactive elements (EREs) in the CD24 promoter inhibits CD24 expression through recruiting estrogen receptor α [23] (Fig. 1).

During the post-transcriptional modification process of CD24, N-glycosylation serves as a chemical signal that regulates protein sorting, thereby controlling the transmembrane transport of CD24 in cancer cells and ultimately affecting the expression level of CD24 on the cell surface. In addition, post-transcriptionally sialylated CD24 can act as the main endogenous ligand for the CD33 family of siglecs, playing a crucial role in protecting the host from inflammatory and autoimmune diseases, metabolic disorders, and respiratory distress, especially during the period of COVID-19, where this protective effect is particularly significant [14].

Epigenetically, non-coding RNAs (ncRNA) can crosslink tumor cells with tumor microenvironment through extracellular vesicles, etc., thereby regulating the expression of CD24. For example, the downregulation of long non-coding RNA (lncRNA) H19 helps to improve the expression of CD24 on the cell surface and increase the invasion ability of tumor [24].

In conclusion, the expression of CD24 is a complex process involving multiple parties, and it is regulated by different biomolecules at various levels of expression. Gaining a deep understanding of the regulation of CD24 expression can provide us with more dimensional insights in terms of mechanism exploration, laying a solid foundation for further studies on the biological functions of CD24.

## Ligands to CD24

CD24, a GPI-anchored protein, is prominently expressed on the surface of various cell types and serves as a critical cell membrane-bound receptor. It is known to engage with a multitude of ligands, thereby mediating a spectrum of biological processes and cellular responses.

### P-selectin

CD24 is a receptor for P-selectin in mouse and human bone marrow and endothelial cells. In human cancer cells, CD24 can interact with P-selectin after proper modification of carbohydrates (such as sialic acid-Lex), encouraging the attachment of ovarian cancer cells to the mesothelium and allowing breast cancer cells to roll and adhere to platelets on endothelial cells [25,26]. This pathway can enhance the metastasis and spread of tumor cells.

### Siglecs

In addition to P-selectin, siglecs, a group of sialic acid-binding receptors on immune cells, are also ligands for CD24. Among them, Siglec-10 (or Siglec G in mouse) is a key immunosuppressive ligand that is expressed on granulocytes, lymphocytes, and monocytes [27]. CD24, Siglec-10 and damage-associated molecular patterns (DAMPs) bind to form trimolecular complexes. The CD24/Siglec-10 axis significantly inhibits tissue damage-induced immune response and mediate immune escape of tumor cells [10,28].

### Pathogen

In addition to its association with tumor cells, CD24 has been found to bind to certain pathogens, including *streptococcus pneumoniae*, which utilizes CD24 as a receptor for adherence and invasion into host cells [28].

The interactions of different ligands with CD24 play a role in a variety of biological processes, and are closely related to the occurrence and development of tumors, as well as immune regulation. Understanding the ligands of CD24 and their biological functions is of great significance for the development of new therapeutic strategies.

## Functions of CD24 in Cancers

Due to differences in glycosylation, CD24 can bind to various ligands and mediate different functions through a variety of molecular signaling pathways, affecting the progression of tumors.

### Intrinsic functions of CD24 in cancers

A range of malignancies, including ovarian cancer, small cell lung cancer, urothelial cancer, liver cancer, cholangiocarcinoma, colorectal cancer, and esophageal squamous cell carcinoma, have been shown to overexpress CD24 in tumor tissues [18,29]. As a GPI-anchored molecule, CD24 mainly locates on the membrane of normal cells. However, in tumor cells, CD24 can be detected on the cell membrane, cytoplasm, and even the nucleus [30,31]. CD24 can alter cancer development by modulating the inner activity of tumor cells.

#### Cancer stem cells

Cancer stem cells (CSCs) are a subgroup of stem cells that are capable of self-renewal, self-differentiating, proliferating indefinitely, evading immune system surveillance, and can resist to chemotherapeutic drugs, among others. CD24 is highly expressed in pancreatic, ovarian, bladder, colorectal and other cancer stem cells.

Pancreatic cancer stem cells exhibit strong chemoresistance and can survive conventional chemotherapy, a trait that underlies the high recurrence and metastasis rates in a large proportion of patients with pancreatic ductal adenocarcinoma who undergo surgical chemotherapy. CD24^+^ pancreatic cancer cell subpopulations exhibit the highest tumorigenic potential, indicating that they may be involved in the development of pancreatic cancer [32]. Research indicates that overexpression of CD24 in pancreatic cancer cell lines promotes cancer stemness and increases the proportion of CSCs, with the specific mechanism potentially related to the activation of Wnt and transforming growth factor-beta (TGF-β) pathways, which in turn promote epithelial-mesenchymal transition (EMT) [33,34]. Similarly, bladder cancer cell lines expressing CD24 exhibit CSC-like characteristics [35]. CD24^+^ ovarian cancer cells display stem-like properties, and they are considered as surface markers for ovarian cancer stem cells [36]. CD24 is also expressed in spheroid cultures of colon cancer CSCs, suggesting that it may serve as a marker for colon cancer CSC populations [37]. In addition to solid tumors, CD24 has been identified as the most highly expressed immune checkpoint in leukemia stem cells (LSCs), and CD24^+^ multiple myeloma (MM) cells constitute a subtype of MM CSCs [38,39].

Therefore, CD24 is expressed in CSCs of both solid and hematological malignancies, making it a potential surface marker for CSCs across various types of malignant tumors and contributing to the occurrence and progression of these cancers.

#### Ligands

Further, CD24 can regulate the malignant behavior of tumor cells, including migration and invasion. CD24 binds to P-selectin, reducing tumor cells adhesion, enhancing endothelial rolling, and promoting metastasis [40,41]. CD24 also acts on integrin subunits, promoting the binding of tumor cells to extracellular matrix components such as collagen, thereby promoting tumor cell deposit and migration [42,43].

Due to the regulation of CD24 on cancer cells, its expression is associated with tumor progress and is used as a stemness marker for cancer [44].

### Extrinsic functions of CD24 in cancers

**Figure 2:**
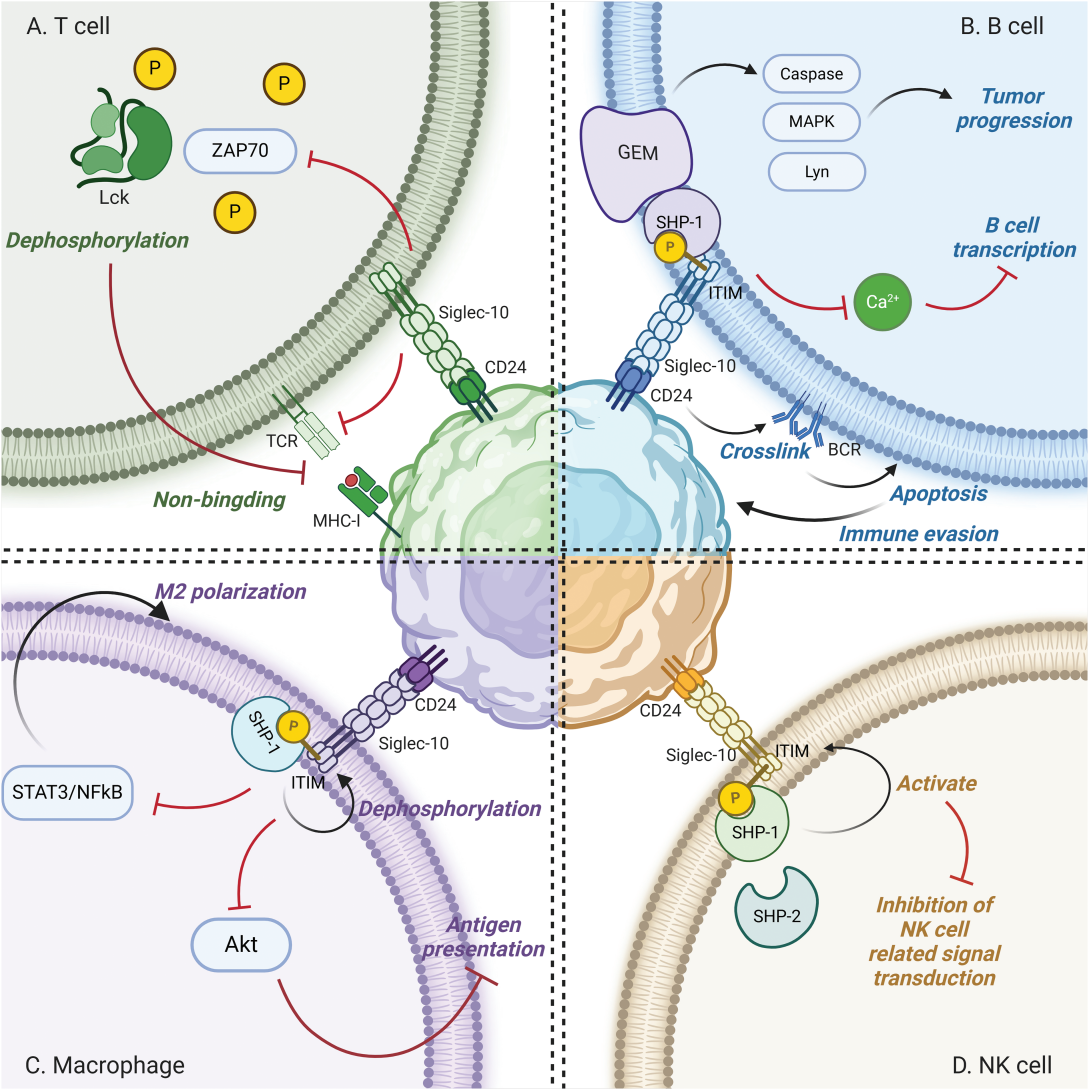
Interactions of CD24 with Immune Cells. (A) In T cells, Siglec-10 prevents the formation of MHC-Ⅰ/peptide complexes by blocking TCR activation and the phosphorylation of T cell receptor-associated kinases Lck and ZAP-70, leading to immunosuppression. (B) B cell-surface Siglec-10 binds CD24, inhibiting calcium signaling and B cell gene transcription. The GEM domain recruits signal molecules, boosts protein kinase activity, and advances tumor growth. Moreover, CD24 induces BCR cross-linking, triggers B cell apoptosis, and aids tumor immune evasion. (C) On macrophages, CD24 binds to Siglec-10, which is phosphorylated by Src family tyrosine kinases, subsequently recruiting and activating proteins containing SH2 domains. This catalyzes the dephosphorylation of tyrosine in the ITIM domain, activating the ITIM and thereby triggering an inhibitory signaling cascade that suppresses the antigen presentation signal transduction of macrophages, promotes M2 polarization of macrophages, inhibits their phagocytic activity, and facilitates the escape of tumor cells from immune system clearance. (D) In the hepatic tumor microenvironment, NK cells express high levels of Siglec-10. Siglec-10 facilitates the expression of ITIM-binding proteins SHP-1 and SHP-2, activates ITIM, and suppresses the function of NK cells in hepatocellular carcinoma. Figure created with BioRender.com.

CD24 can also rely on altering the activity of external immune cells to regulate the progression of cancer. CD24 primarily modulates various immune cells to help cancer cells evade immune surveillance by binding to siglec10 on immune cells, contributing to the development of cancer (Fig. 2).

#### T cells

CD24 also plays a key role in adaptive immune cell function regulation. In T cells, as a result of blocking T cell receptor (TCR) activation and the phosphorylation of the T cell receptor-associated kinases Lck and ZAP-70, Siglec-10 prevents T cells from forming MHC-I/peptide complexes, leading to immunosuppression [45,46].

#### B cells

In B cells, Siglec-10 inhibits calcium signaling by recruiting proteins with ITIM domains such as Src homology 2 domain-containing protein tyrosine phosphatase 1(SHP-1) and growth factor receptor-bound protein 2 (Grb2), and suppresses the expression of transcription factors, thereby inhibiting the proliferation of B cells [47]. Additionally, the cross-linking of Siglec-10 on the surface of B cells with CD24 inhibits BCR-mediated signal transduction. The glycolipid-enriched membrane (GEM) domain serves as a platform for recruiting various signal transduction molecules, and the activities of Src family tyrosine kinase member Lyn, mitogen-activated protein kinase (MAPK), cysteine proteases, caspases, and other protein kinases are enhanced, promoting the development of tumors [48]. Further research indicates that CD24, during the pre-B phase, activates multiple extracellular signal-regulated kinases through the cross-linking of BCR precursors. Specifically, the cross-linking of CD24 with ERK38 initiates inhibitory signals, mediating the apoptosis of human precursor B cells and triggering the immune escape of tumors [49,50].

#### Macrophages

CD24 is a “don’t eat me” signal that has inhibitory effects in tumor immunity by binding to Siglec-10 on macrophages. On macrophages, Siglec-10 has two immunoreceptor tyrosine-based inhibitory motifs (ITIMs) in its cytoplasmic tail. When the IgV domain of Siglec-10 binds to the sialic acid moiety located in the extracellular domain of CD24, the Src family tyrosine kinases in the cytoplasmic tyrosine signaling group are activated by the ITIMs. Once phosphorylated, ITIMs can recruit and activate proteins containing SH2 domains, specifically two SH domain-containing protein phosphotyrosine phosphatases called SHP-1 and SHP-2, or suppressor of cytokine signaling 3 (SOCS3) [51,52]. SHP-1 selectively binds to the phosphorylated tyrosine residues within the ITIM domain inside the cell, then catalyzes their dephosphorylation, blocking downstream signal transduction pathways and playing a negative regulatory role. The activation of SHP-1 significantly inhibits Akt2, thereby reducing the production of pro-inflammatory cytokines and the expression of antigen presentation mechanisms in macrophages [53]. The activation of SHP-1 can also downregulate the expression of colony-stimulating factor 1 receptor (CSF1R) and inactivate the STAT3/nuclear factor kappa B (NFκB) signaling pathway, ultimately promoting the polarization of macrophages towards the M2 phenotype and exhibiting an anti-inflammatory profile [54]. Research has found that inhibiting SHP-1 can effectively enhance the immune surveillance function of macrophages and NK cells against leukemia stem cells (LSC) [55]. The interaction of CD24 with Siglec-10 triggers a SHP-1-mediated inhibitory signaling cascade that inhibits phagocytosis of macrophages [56].

#### NK cells

Siglec-10 is highly expressed on NK cells in the liver tumor microenvironment. Siglec-10 promotes the expression of ITIM-binding protein SHP-1, 2, activates ITIM, and inhibits the role of NK cells in hepatocellular carcinoma [57,58].

CD24 can promote the progression of cancer by exerting both intrinsic and extrinsic functions. On one side, it enhances the proliferation and invasiveness of cancer cells; on the other side, it decreases the clearance function of immune cells. Together, these effects facilitate the immune evasion of tumor cells. This bidirectional regulation provides us with corresponding insights. When targeting CD24 for cancer treatment, we can not only directly block CD24 but also target its ligand siglec-10, as well as enhance the functions of immune cells for treatment, or even combine these approaches. To date, many studies have made progress within these complex signal pathways, but more specific mechanisms still require further research.

### CD24 is associated with poor prognosis

Growing clinical evidence suggests that patients expressing high levels of CD24 are at a higher risk of disease progression and cancer-related death. In ovarian cancer, Kristiansen et al. found CD24 expression in 59% of cases, which was associated with shortened overall survival [59]. Similarly, in colorectal cancer, abnormally strong cytoplasmic CD24 expression was seen in 24% of cases and was significantly associated with shortened distant metastasis-free survival [60]. In non-small cell lung cancer, 87 out of 267 cases (33%) showed high expression of CD24, and these patients had a higher risk of disease progression and mortality [61].

In summary, CD24 has been confirmed to be overexpressed in a variety of cancers, including head and neck cancer [62], breast cancer [63,64], lung cancer [65], colorectal cancer [66], pancreatic cancer [67], hepatocellular carcinoma [68], ovarian cancer [69], urothelial carcinoma [70], prostate cancer [71] and hematological malignancies [72,73]. Both laboratory and clinical data indicate that the overexpression of CD24 is often associated with poor prognosis. This evidence suggests a close link between CD24 and resistance to cancer treatment. Below, we will further explore the current preclinical evidence of CD24 in promoting cancer drug resistance and progression. These preclinical studies also provide insights into understanding the underlying mechanisms.

## The Role of CD24 in Chemotherapy Resistance

Apart from its essential role in regulating tumor progression and immune evasion, CD24 is found to be involved in chemotherapy resistance.

### Head and neck cancer

CD24-positive head and neck squamous cell carcinoma (HNSCC) cell lines maintain a high capacity for self-renewal, exhibit stem-like characteristics, and possess resistance to cisplatin. In CD24-rich cell lines, treatment with a monoclonal antibody to block CD24 can reduce cisplatin resistance. It is currently believed that CD24 can alter the expression levels of DNA repair genes (such as L1CAM and NBS1) on the cell membrane by acting as a controller of lipid rafts, to counteract the toxicity and damage to cancer cells induced by cisplatin [74]. High expression of CD24 in locally advanced oral squamous cell carcinoma also leads to poor response to chemotherapy and poor prognosis [62].

### Pancreatic cancer

An increased proportion of CD24^+^ cells has been identified within the context of gemcitabine-resistant pancreatic cancer cells. Experimental evidence suggests that there is an upregulation of the Gli-SOX2 signaling cascade in pancreatic cancer cell lines exhibiting resistance to gemcitabine. Furthermore, the overexpression of GLI transcription factors has been correlated with elevated levels of CD24, a marker indicative of tumor-initiating cells (TICs), thereby contributing to the accumulation of CD24 within the subpopulation of pancreatic cancer stem cells. This molecular interplay may provide a potential therapeutic target for overcoming chemoresistance in pancreatic cancer [75].

### Ovarian cancer

Ovarian cancer cells, delineated from a homogenous cell line and stratified based on CD24 expression, demonstrate a dichotomy in their therapeutic responses. Specifically, the CD24-positive subpopulation of these cells exhibits enhanced resistance to the chemotherapeutic drugs cisplatin and doxorubicin, while paradoxically displaying increased susceptibility to lysis mediated by natural killer (NK) cells. This paradoxical phenotype suggests that NK cell-based immunotherapeutic interventions could be a promising avenue to surmount chemoresistance observed in CD24-positive ovarian cancer cells [76].

### Leukemia

Within the context of leukemia, the therapeutic resistance observed is underpinned by the presence of LSCs. CD24, as an immune checkpoint of LSCs, promotes tumorigenesis and therapeutic resistance through the regulation of the Wnt-β-catenin and PI3K-Akt pathways [38].

### Retinoblastoma

In retinoblastoma (RB), CD24 recruits lipid rafts and activates the PTEN/AKT/mTORC1 signaling pathway. The downstream activation of autophagy reduces the sensitivity of RB to vincristine (VCR), leading to chemoresistance [77].

### Melanoma

In melanoma, SOX2 mediates the upregulation of CD24, which promotes the adaptability and resistance of melanoma cells to B-Raf proto-oncogene (BRAF) inhibitor targeted therapy. Moreover, knocking down CD24 in SOX2-overexpressing drug-resistant cells can restore their sensitivity to BRAF inhibitors [78].

In summary, the aforementioned preclinical studies revealed a profound connection between the overexpression of CD24 and chemoresistance, with the complex molecular biology pathways pointing the way for subsequent mechanistic research.

## The Role of CD24 in Radiotherapy Resistance

The relationship between CD24 and radiotherapy resistance is less studied and remains unclear. Study shows among the radiation-resistant cervical cancer cells, most are CD44^+^/CD24^+^ cells, which share other CSC characteristics, such as B lymphocytoma 2 (Bcl-2), increased expression of survival proteins, and show greater tumorigenicity [79]. CD24 gene expression was higher in cervical cancer samples compared to neighboring non-cancerous tissues. It was also observed that CD24 promoted the proliferation and invasion of cervical cancer cells *in vitro*, inhibited their apoptosis, and helped cervical cancer cells to develop radiotherapy resistance. In patients with cervical squamous cell carcinoma who received postoperative radiotherapy, patients with a high percentage of CD24^+^ tumor cells were found to have significantly lower distant metastasis-free survival and overall survival than patients with a high percentage of CD24^−^ cells.

In patients with locally advanced HNSCC receiving accelerated platinum-based radiotherapy, the widespread presence of CD24^+^ cancer cells is directly associated with an increased proliferation index and is related to a significantly poorer local progression-free interval. High expression of CD24 is also slightly associated with larger tumor size, indicating resistance to radiotherapy [80].

In pancreatic cancer, the resistance of tumors to radiotherapy is closely related to the presence of apoptosis-resistant CSCs, and CD24 is also one of the biomarkers of pancreatic cancer stem cells. Experiments have shown that radiotherapy-resistant pancreatic cancer cells express higher levels of CD24 and have stronger tumorigenicity both *in vitro* and *in vivo* [81].

In conclusion, overexpression of CD24 can induce radiotherapy resistance, affect the efficacy of radiotherapy, and is not conducive to the prognosis of patients. But the specific mechanism needs further study.

## The Mechanisms of CD24-Induced Chemoradiotherapy Resistance

CD24 mediates radiotherapy and chemotherapy resistance by affecting the intrinsic functions of tumor cells and promoting tumor occurrence and progression. On the other hand, CD24 affects the function of the immune system, promotes a series of immunosuppression-related responses, helps tumor cells escape immunity, and mediates radiotherapy and chemotherapy resistance.

### Intrinsic mechanisms in CD24-mediated chemoradiotherapy resistance

#### p38MAPK pathway

**Figure 3:**
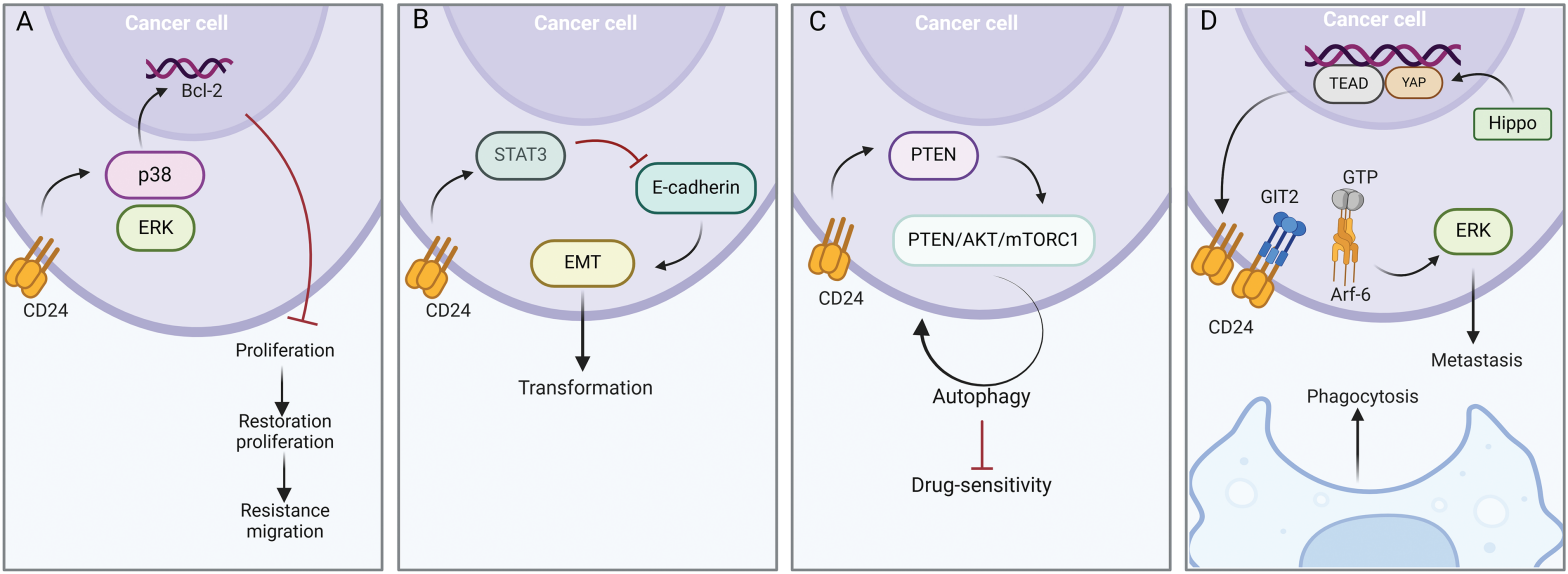
Intrinsic mechanisms in CD24-mediated chemoradiotherapy resistance. (A) p38 MAPK pathway. In the CD24-dependent MAPK pathway, the sustained activation of the ERK and p38 MAPK genes promotes the expression of the Bcl-2, which in turn enhances the drug resistance and migration capabilities of tumor cells. (B) STAT3 pathway. The expression of CD24 can activate the phosphorylation of STAT3, inhibit E-cadherin, and regulate EMT, which leads to the loss of intercellular adhesion in tumor tissue and promotes tumor metastasis. (C) PI3K-AKT pathway. CD24 facilitates chemoresistance in cancer cells by recruiting PTEN to the lipid raft domain and activating autophagy through the PTEN/AKT/mTORC1 signaling pathway. (D) The activation of the Hippo signaling enhances the activity of transcription factors YAP and TEAD, promoting the expression of the CD24 gene. High expression of CD24 competitively binds to GIT2 with Arf-6, increasing the stability of Arf6/GTP, activating the downstream ERK pathway, and promoting tumor cell metastasis and macrophage phagocytosis. Figure created with BioRender.com.

CD24 can activate the proliferation of cancer cells through the CD24-dependent MAPK pathway, in which extracellular regulatory protein kinase (ERK) and p38 mitogen-activated protein kinase (p38 MAPK) are key factors. Sustained activation of the p38 gene promotes the expression of the Bcl-2 gene, a phenotype that inhibits cell proliferation temporarily. Cancer cells that are suppressed from proliferating will resume proliferation after a few weeks and have greater drug resistance and migration. In breast cancer, siRNA-mediated CD24 down-expression inhibited phosphorylation of p38, thereby improving the efficacy of doxorubicin therapy [38,82] (Fig. 3A).

#### STAT3 pathway

The expression of CD24 can also regulate the phosphorylation-related reactions in the STAT3 pathway, promoting the activation of downstream STAT3 signaling, thereby inhibiting E-cadherin and enhancing EMT. EMT is closely related to the stemness and drug resistance of tumors. A study on pancreatic cancer stem cells with high expression of CD24 has shown that EMT mediates the resistance of cancer cells to gemcitabine [83,84] (Fig. 3B).

#### PI3K-AKT pathway

CD24 may also induce chemoresistance via the PI3K-AKT pathway. CD24 recruits PTEN to the lipid raft domain and activates autophagy by regulating the PTEN/AKT/mTORC1 signaling pathway. Autophagy can be used as a protective mechanism mediating chemotherapy resistance of cancer cells, reducing the sensitivity of cancer cells to drugs. In a study on RB, knockdown of CD24 was found to eliminate autophagy through the PTEN/Akt/mTORC1 pathway and significantly improved the inhibitory effect of vincristine on RB cells, enhancing the efficacy of chemotherapy [77,85]. In hepatocellular carcinoma, overexpression of CD24 leads to increased production of PP2A protein, induces inactivation of the AKT/mTOR pathway, elevates autophagy levels, and results in resistance to sorafenib [86,87] (Fig. 3C).

#### Hippo-YAP-CD24-Arf6-ERK pathway

The Hippo-YAP signaling transduction pathway is a potent oncogenic initiator in esophageal squamous cell carcinoma (ESCC) [88]. Unlike the Siglec pathway, YAP directly regulates CD24 transcription through the binding of transcriptional enhanced associate domain (TEAD) to the CD24 promoter region. Studies have shown that activation of YAP induces upregulation of CD24 expression and significantly inhibits macrophage phagocytosis, and this effect can be reversed by inhibition of CD24. Highly expressed CD24 competitively binds to ADP-ribosylation factor 6(Arf6) with G protein-coupled receptor kinase-interactor 2(GIT2) and stabilizes Arf6-GTP to activate subsequent ERK pathways, thereby significantly promoting tumor metastasis and 5-FU chemoresistance [89] (Fig. 3D).

The Hippo-YAP pathway has also been shown to indirectly regulate CD24 expression through the transcription factor SOX4 in hepatocellular carcinoma and promote tumor progression [90]. This high expression of CD24 also promotes the development of chemoresistance in hepatocellular carcinoma [86].

### Extrinsic mechanisms in CD24-mediated chemoradiotherapy resistance

In addition to directly acting on tumor cells, CD24 can also interact with Siglec-10 to regulate the physiological functions of immune cells and affect the immune response to tumors.

In ovarian cancer, TAMs can release extracellular vesicles (EVs), in which the expression of GATA-binding protein 3 (GATA3) is highly upregulated within the EVs and is transferred into ovarian cancer cells. The upregulation of GATA3 leads to an increase in the expression of CD24, which in turn enhances the expression of Siglec-10 on immune cells. This increase in Siglec-10 expression augments the apoptosis rate of T cells and delays T cell activation, ultimately facilitating the immune evasion of OC cells and their resistance to cisplatin, thereby exacerbating the progression of ovarian cancer [91].

Overall, the CD24-mediated pathways played a role in both innate and adaptive immunity, inducing chemoradiotherapy resistance in tumor cells, and the specific mechanisms still require further exploration. Considering the multifaceted characteristics of CD24 in promoting cancer progression, facilitating immune evasion, and inducing resistance to radiochemotherapy, we believe these mechanisms are closely interconnected. Identifying the most critical point within this network is the direction that needs to be investigated next, and it is also the challenge that must be overcome for CD24 to be applied in clinical precision therapy. Metabolomics is a branch of omics that involves the quantitative analysis of all metabolites present in an organism to study their relative relationships during physiological and pathological changes. With the continuous advancement of technology, it may be possible to utilize metabolomics techniques to investigate metabolites that have a causal relationship with the mechanisms of chemoresistance induced by CD24, which represents an entirely new direction [92].

## Targeting CD24 for Cancer Therapies: Preclinical Evidence

**Figure 4:**
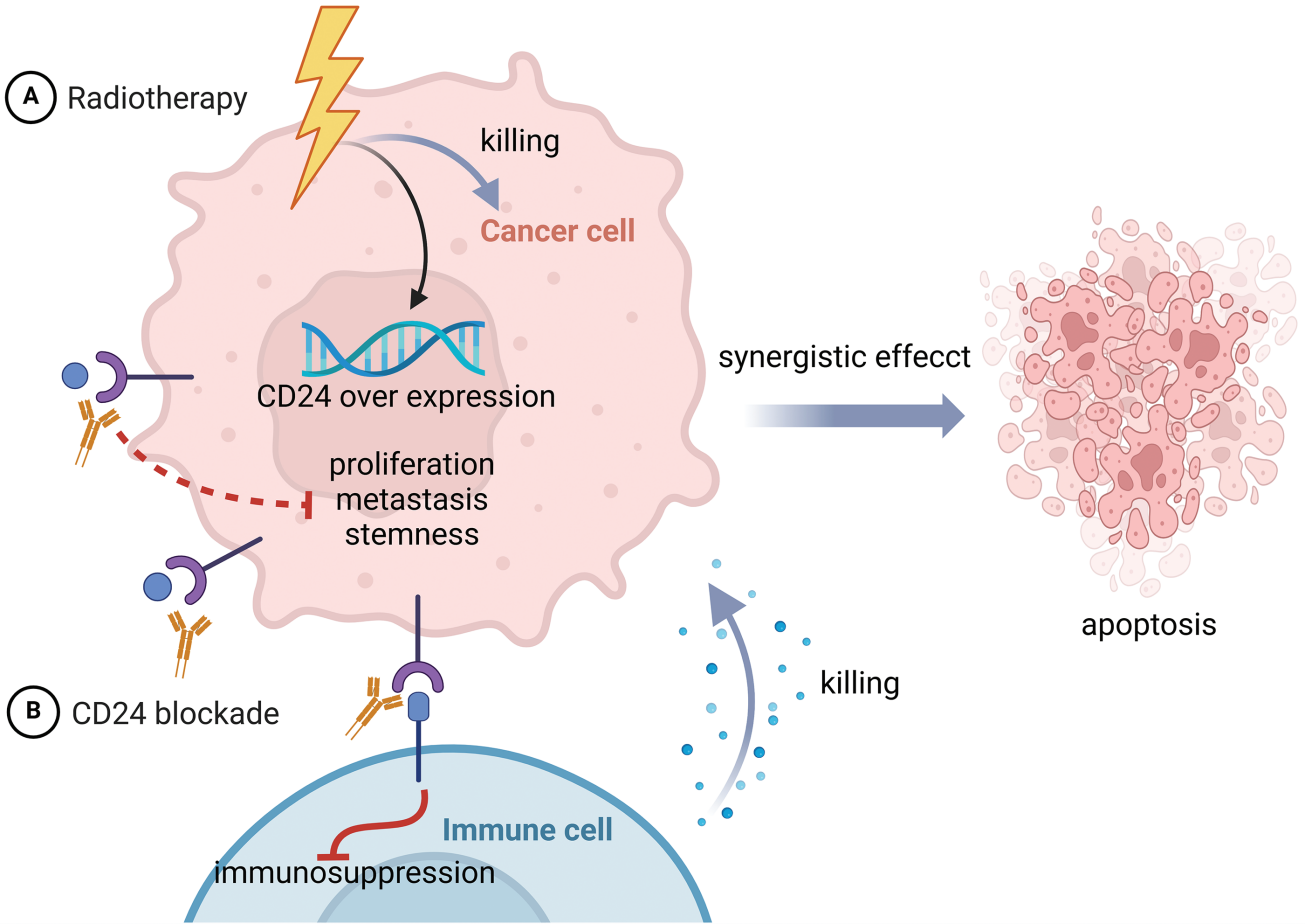
Radiotherapy combined with CD24 blockade for cancer treatment. (A) Radiotherapy directly damages tumor cells but also upregulates CD24 expression, promoting immune evasion. (B) CD24 blockade therapy can counteract the effects of radiation therapy-induced upregulation of CD24. The two work synergistically to enhance immune cell-mediated tumor cytotoxicity and improve therapeutic efficacy. Figure created with BioRender.com.

In view of the high expression of CD24 in cancer cells and the role of inducing resistance in chemoradiotherapy, CD24 is considered a promising new target for cancer treatment. Increasing effort has been made to explore whether CD24-targeted therapy combined with chemoradiotherapy can achieve better therapeutic effects (Fig. 4A,B).

### Anti-CD24 antibodies

CD24 induces high expression of pluripotent stem cells in malignant mesothelioma. The CD24 antibody inhibited the growth of MM cells and blocked the progression of MM [93]. These suggested that CD24 could be a new target for mesothelioma therapy. In lung and ovarian cancer models, targeting CD24 with SWA11 monoclonal antibody effectively delayed the growth of xenografts, reduced blood vessel generation, increased macrophage infiltration of tumor tissue, rendering tumor cells more sensitive to gemcitabine chemotherapy [94,95].

IMM47 is a humanized monoclonal antibody targeting CD24, which works by blocking the CD24/Siglec-10 interaction through macrophage antigen presentation. IMM47 can be used as a monotherapy or in combination with drugs such as tislelizumab, demonstrating excellent antitumor efficacy. Additionally, IMM47 significantly binds to granulocytes but not to human red blood cells, offering good safety [96].

### Engineered anti-CD24 CAR-NK cells

Klapdor et al. utilized an anti-CD24 antibody SWA11, which has high affinity, potent cytotoxic activity, and low off-target effects, from which they selected a single-chain variable fragment (scFv). This fragment was cloned into a chimeric antigen receptor (CAR) backbone targeting CD24 and then transduced into the human NK cell line NK-92 using a lentivirus. The CAR-engineered NK-92 cells demonstrated specificity and potent cytotoxic activity against antigen-presenting ovarian cancer cell lines as well as primary ovarian cancer cells of significance [97,98].

### Molecular radiotherapy targeting CD24

In a study of malignant mesothelioma, CD26/CD24-deficient cell lines grew slower, were less invasive but more sensitive to drug therapy. It suggested that molecular radiotherapy targeting CD26 and CD24 may be a promising approach to targeted therapy against malignant mesothelioma CSCs [93].

### Blocking peptides

Recently scientists discovered a novel dual-targeted peptide that blocks CD24/Siglec-10 and PD-1/PD-L1 interactions, and demonstrated that radiation therapy (RT) can recruit tumor immune cells and upregulate PD-L1 expression, while CD24/Siglec-10 blocking peptide (CSBP) could suppress PD-1/PD-L1 expression. Combining RT with CSBP therapy can improve anti-tumor effectiveness [99].

### Antibody-drug conjugates

Antibody-drug conjugates (ADCs) are a novel targeted drug delivery system that link antibodies to active cytotoxic drugs and deliver them to tumors [100]. Compared to other cancer therapies, ADCs enhance the therapeutic index by limiting the exposure of cytotoxic drugs to normal cells, thereby efficiently suppressing off-target toxicity and reducing the toxicity of cytotoxic drugs to other healthy tissues throughout the body [101].

A drug conjugate of nitric oxide (NO) with anti-CD24 antibodies can selectively and efficiently inhibit liver cancer. Preclinical studies have indicated that it significantly increases the release of NO from hepatocellular carcinoma cells, induces apoptosis, and inhibits tumor growth [102,103].

In summary, drugs targeting CD24 show promising prospects for clinical application. However, it should not be overlooked that due to the expression of CD24 on the surface of normal cells and potential off-target effects, it may cause a certain degree of damage to non-tumor cells in the body. Currently, ADC therapy has achieved certain breakthroughs in tissue selectivity, and further optimizing its structure to reduce the toxic and side effects on patients is a direction for future research. The latest research indicates that the genetic ablation of CD24 and Siglec-10 is an effective method for targeting tumor cells and enhancing the phagocytic action of macrophages. However, the instability of RNA interference (RNAi) technology and related ethical issues require researchers to approach such therapies with caution [104].

## Targeting CD24 for Cancer Therapies: Clinical Explorations

Up to now, two clinical trials utilizing CD24-targeted immunotherapy in cancer patients have been completed. A single-arm phase I/II trial investigated the efficacy of treatment with specific anti-CD21 and anti-CD24 murine monoclonal antibodies (MoAbs) in patients who developed aggressive B-cell lymphoproliferative disorder (BLPD) following bone marrow transplantation (BMT) or organ transplantation [105]. BLPD, a rare but extremely severe complication post-transplant, may arise due to cytotoxic T-cell deficiencies. This open-label, multicenter trial enrolled 58 patients who received a 10-day course of combined therapy with 0.2 mg/kg/day of specific anti-CD21 and anti-CD24 murine MoAbs [19]. The treatment was well-tolerated, with common adverse events including grade 3 or higher transient neutropenia (42%) and grade 2 fever (22%). The study achieved significant therapeutic outcomes, with 36 patients (61%) achieving complete remission, and only 3 of these initial responders (8%) experienced relapse [106]. The results of this trial suggest a correlation between complete remission and survival rates, and compared to other methods for treating BLPD, including chemotherapy, the combined targeted therapy is an effective and straightforward treatment option that warrants further efficacy studies and comparisons.

The second trial was a single-center exploratory study assessing the efficacy of DC–CIK (dendritic cell–cytokine-induced killer cell) immunotherapy in patients with primary liver cancer who underwent radical resection. The trial noted elevated expression of CD24 in primary liver cancer and incorporated CD24 peptides into the treatment protocol. Follow-up assessments over 1–4 years revealed changes in immune cell counts in patient serum, indicating modulation of immune balance. The 4-year survival rates for patients who received two and four immunotherapy sessions were 47.1% and 52.6%, respectively. Compared to historical data, this therapy delayed recurrence, improved survival rates, and demonstrated safety [107]. However, due to the limited follow-up period and sample size (36 patients), further clinical evidence is required to validate these results.

Other clinical trials focusing on CD24-targeted immunotherapy are underway. IMM47, the first anti-CD24 drug declared in China, has published preclinical evidence [96], and its phase I clinical trial in Australia has successfully enrolled the first subject, officially entering the clinical research phase. NCT06028373 is a phase I PERFORM study designed to assess the safety and preliminary efficacy of ATN-031 in treating patients with advanced solid tumors or B-cell non-Hodgkin lymphoma (B-NHL). A phase I clinical trial of the anti-CD24 monoclonal antibody NXA01 has also been approved, and it is noteworthy for its ability to selectively target and kill CD24-positive tumor cells; the outcomes of this trial are eagerly anticipated.

CD24, as a novel target of significant interest, is also explored in other trial directions. Multiple trials have demonstrated that CD24 can serve as a cancer biomarker. For instance, a phase II trial involving 257 patients indicated that CD24-type alanine/valine single-nucleotide polymorphisms could predict pathological complete response (pCR) following neoadjuvant chemotherapy in primary breast cancer and modulate the host’s antitumor immune response. Takahashi et al. analyzed CD24 expression in gastric adenocarcinoma tissues and correlated it with clinical parameters and patient survival rates. The results showed that high CD24 expression was closely associated with the depth of gastric cancer invasion, high pathological stage, and poor prognosis (NCT01214512). CD24 can act as a specific cancer biomarker, providing a theoretical basis for targeted therapy of tumor cells and serving as a reference for prognosis and recurrence monitoring. Additionally, the relationship between CD24 and side effects of tumor treatment is also under clinical trial investigation. A phase I clinical trial assessed the safety and tolerability of intravesical injections of CD24Fc fusion proteins, showing a low incidence of treatment-emergent adverse events (TEAEs), indicating a high safety profile for CD24Fc injections at appropriate doses. CD24Fc can also mitigate autoimmune and inflammatory responses during cancer treatment, shortening the recovery time of immune-related adverse events (irAEs) and reducing the severity of immunotherapy side effects (NCT04552704) (Table 1).

**Table 1:** Clinical trials of CD24-targeted therapies

NCT	Status	Conditions	Interventions	Simple size	Primary outcomes
NCT04060407	Withdrawn	Metastatic melanoma	CD24Fc, Ipilimumab and Nivolumab	0	TRAE*
NCT04552704	Terminated	Advanced malignant solid neoplasm	CD24Fc and Placebo	3	AE*
NCT05888701	Recruiting	Mantle-cell Lymphoma and B cell chronic Lymphocytic Leukemia	*In vitro* experiments of patient-derived monocyte systems	30	Rate of phagocytosis
NCT01214512	Completed	Colorectal cancer	blood-sample based diagnostic assay	229	Colonoscopy and CD24 assay correlation
NCT02650895	Completed	Healthy volunteers	Efprezimod alfa and Saline	40	TEAEs*
NCT00485979	Completed	Primary breast cancer	CD24-type Ala/Val single-nucleotide polymorphisms	257	pCR*

Note: *: TRAE, treatment-related adverse events; AE, adverse events; TEAEs, treatment-emergent adverse events; pCR, pathological complete response.

Targeting CD24 represents a promising new avenue in cancer immunotherapy, with unique advantages, especially in overcoming immune evasion. However, this approach faces significant challenges, including limited clinical data and potential off-target effects. In comparison, established therapies such as cancer immune checkpoint inhibitors (ICIs) and CAR T-cell therapies have shown tremendous efficacy, but they also have their own limitations, such as adverse events and high costs. Cancer vaccines, while safe and preventive in potential, still face difficulties in terms of therapeutic effects for diagnosed cancers. In the urgent need to combat tumors, accelerating clinical research on CD24 is crucial, whether it is developing new antibodies to more precisely target tumor cells, studying the synergistic combination of anti-CD24 with other immunotherapies to improve overall treatment outcomes, or exploring the combination of anti-CD24 with radiochemotherapy to combat tumor resistance, these are all directions and pathways worth exploring.

## Prospectives and Conclusions

In-depth studies found that CD24 affects the behavior of tumor cells and the function of immune cells, induces the resistance to radiotherapy and chemotherapy, and promotes the progression and poor prognosis of cancer. With increased understanding of cancer immunological mechanisms, particularly the role of “don’t eat me” CD24/Siglec10 signaling, we propose that CD24 blockade is a promising strategy for cancer treatment. Due to the high expression of CD24 in tumor cells and its close association with mechanisms of resistance to radiochemotherapy, targeting CD24 can precisely deliver cancer cell killing and suppress the emergence of chemoradiotherapy resistance. Ongoing clinical trials have shown promising results in the development of CD24 targeted drugs. To date, monoclonal antibodies, antibody–drug conjugates, and cellular therapies such as CAR-NK have demonstrated significant efficacy. It should be noted that monotherapies often lead to resistance in cancer treatment. Therefore, we believe that CD24 targeting can be combined with other forms of cancer therapies, including traditional chemotherapy, radiotherapy, or other immunotherapies targeting different pathways or mechanisms, potentially yielding synergistic effects. In current clinical practice, novel treatment regimens combining conventional chemotherapy with ICI-based immunotherapy have been widely applied and have shown significant efficacy. Considering the positive results of CD24 in preclinical and clinical studies, targeting CD24 in combination with chemotherapy is a hopeful strategy for cancer treatment.

However, current studies targeting CD24 also have certain challenges. Firstly, the precise targeting of the drug affects its efficacy, and since CD24 is expressed in many tissues, further exploration is needed to achieve accurate targeting of CD24 in tumor tissues. Moreover, the lack of mature biomarkers to predict response to anti-CD24 antibody therapies is also a challenge, which can pose potential dangers to patients. Additionally, there are concerns regarding drug safety. The expression of CD24 varies between human and mouse normal tissues, and after the results of preclinical trials are known, we must still consider the potential toxicity of CD24 targeting and its unknown adverse consequences for humans. This issue might be addressed using human CD24 transgenic mice. In combination therapy, it is also important to evaluate whether the efficacy overweighs the potential double drug toxicity. At present, there are few clinical trials on CD24 blocking combined with chemoradiotherapy, and more clinical data are needed to convince its safety and efficacy. Cautious evaluation should be carried out before such treatment is administered to patients. In the future, precision drug delivery and improvements in treatment strategies will help to enhance the effectiveness of treatment and reduce the toxic effects of drugs.

## Data Availability

The data that support the findings of this study are available from the corresponding author upon reasonable request.

## Abbreviations

ADCs: Antibody-drug conjugates
Akt: Protein kinase B
Arf6: ADP-Ribosylation Factor 6
Bcl-2: B lymphocytoma 2
BCR: B cell receptor
BRAF: B-Raf proto-oncogene
CAR: Chimeric antigen receptor
CLL: Chronic lymphocytic leukemia
CSBP: CD24/Siglec-10 blocking peptide
CSCs: Cancer stem cells
CSF1R: Colony-stimulating factor 1 receptor
DAMPs: Damage-associated molecular patterns
EMT: Epithelial mesenchymal transformation
EREs: Estrogen-reactive elements
ERK: Extracellular regulatory protein kinase
ESCC: Esophageal squamous cell carcinoma
EVs: Extracellular vesicles
GATA3: GATA-binding protein 3
GEM: Glycolipid-enriched membrane
GIT2: G protein-coupled receptor kinase-interactor 2
Gli2: GLI family zinc finger 2
GPI: Glycosyl-phosphatidylinositol
Grb2: Growth factor receptor-bound protein 2
GTP: Guanosine triphosphate
HIFs: Hypoxia-inducible factors
HNSCC: Head and neck squamous cell carcinoma
ICIs: Immune checkpoint inhibitors
irAE: Immune-related adverse events
ITIMs: Immunoreceptor tyrosine-based inhibition motifs
LILRB-1: Leukocyte Immunoglobulin-like Receptor Subfamily B Member 1
lncRNA: Long non-coding RNA
LSCs: Leukemia stem cells
MAPK: Mitogen-activated protein kinase
MCL: Mantle cell lymphoma
MDSCs: Myeloid-derived suppressor cells
MHC-I: Major histocompatibility complex class I
MM: Multiple myeloma
mTORC1: Mechanistic target of rapamycin complex 1
ncRNA: Non-coding RNAs
NF-κB: Nuclear factor kappa B
OC: Ovarian cancer
PBL: Peripheral blood leukocytes
pCR: Pathological complete response
PI3K: Phosphatidylinositol-3-kinase
PP2A: Protein phosphatase 2A
PTEN: Phosphatase and tensin homolog
RB: Retinoblastoma
RNAi: RNA interference
RT: Radiation therapy
scFv: Single-chain variable fragment
SHP-1: Src homology 2 domain-containing protein tyrosine phosphatase 1
SIRP: Signal regulatory protein
SOCS3: Suppressor of cytokine signaling 3
SOX2: Sex determining region Y-box 2
STAT3: Signal transducer and activator of transcription 3
TAMs: Tumor-associated macrophages
TEAD: Transcriptional enhanced associate domain
TEAEs: Treatment-emergent adverse events
TGF-β: Transforming growth factor-beta
TICs: Tumor-initiating cells
TME: Tumor microenvironment
Tregs: Regulatory T cells
VCR: Vincristine
YAP: Yes-associated protein
